# Loss of PTEN as an Independent Poor Prognosis Indicator in Lung Adenocarcinoma, but Not in Squamous Cell Carcinoma, Is Associated with an Immunosuppressive Tumor Microenvironment and Distinct Co-Mutational Profiles

**DOI:** 10.3390/medsci14020254

**Published:** 2026-05-14

**Authors:** Maeva Houry, Shannon J. Silva, Maider Artola, Carmen Behrens, Katerina Politi, Ignacio Wistuba, Luis Montuenga, Francisco Exposito, Alfonso Calvo

**Affiliations:** 1 Program in Solid Tumors, Center for Applied Medical Research (CIMA), Cancer Center Clinica Universidad de Navarra (CCUN), University of Navarra, Avenida de Pío XII, 55, 31008 Pamplona, Spain; mhoury@unav.es (M.H.);; 2 Department of Pathology, Anatomy and Physiology, School of Medicine, University of Navarra, 31008 Pamplona, Spain; martolalarr@unav.es; 3 Department of Pathology, Yale University School of Medicine, New Haven, CT 06520, USA; shannon.silva@yale.edu (S.J.S.); francisco.exposito@yale.edu (F.E.); 4 Department of Medicine (Medical Oncology), Yale University School of Medicine, Yale Cancer Center, New Haven, CT 06520, USA; 5 Department of Translational Molecular Pathology, UTHealth Houston Graduate School of Biomedical Sciences, The University of Texas MD Anderson Cancer Center, Houston, TX 77030, USA; 6 CIBERONC, ISCIII, 28029 Madrid, Spain; 7 IDISNA, 31008 Pamplona, Spain

**Keywords:** lung adenocarcinoma, lung squamous carcinoma, prognosis, PTEN, mutation patterns, immune populations

## Abstract

**Background/Objectives:** Non-small cell lung cancer (NSCLC) comprises biologically heterogeneous tumors, primarily lung adenocarcinoma (LUAD) and lung squamous cell carcinoma (LUSC), which differ in genomic landscape and clinical behavior. The tumor suppressor PTEN is a key negative regulator of the PI3K/AKT/mTOR pathway and is frequently inactivated in NSCLC through genetic and non-genetic mechanisms. Although reduced PTEN expression has been associated with poor outcomes in lung cancer, its prognostic relevance across these histological subtypes remains unclear. **Methods:** Here, we investigated the prognostic significance of PTEN in NSCLC subtypes using a multi-level approach combining protein, transcriptomic, and genomic analyses. PTEN protein expression was evaluated by immunohistochemistry in a tissue microarray from resected NSCLC patients, and findings were validated using publicly available datasets including TCGA-RPPA, GEO/EGA-based transcriptomic cohorts, and large genomic resources. In parallel, mutational landscapes and co-mutation patterns were analyzed in several independent datasets, and tumor immune microenvironment composition was inferred using CIBERSORT deconvolution analysis. **Results:** Low PTEN protein and mRNA levels, as well as *PTEN* mutations, were consistently associated with significantly worse overall survival (OS) in LUAD but not in LUSC. Multivariable Cox regression analysis confirmed PTEN as an independent prognostic factor in LUAD. Although *PTEN* mutations were more frequent in LUSC, they showed no prognostic value in this subtype. Co-mutation analyses revealed recurrent *PTEN* partnerships with *TP53*, *EGFR*, and *APC* in LUAD, with *PTEN-TP53* co-alterations enriched in metastatic disease. Immune deconvolution demonstrated that PTEN-low LUAD tumors were characterized by an immunosuppressive microenvironment, including increased T regulatory cells and reduced inflammatory immune populations. Notably, increased M2-like macrophages were associated with shorter OS in PTEN-low LUADs, whereas a high number of total macrophages (CD68+ cells) emerged as an independent predictor of more favorable OS. **Conclusions:** Collectively, these results identify PTEN loss as a subtype-specific prognostic biomarker in LUAD and link its deficiency to immunosuppressive tumor microenvironment remodeling.

## 1. Introduction

Non-small cell lung cancer (NSCLC) is a biologically heterogeneous cancer that includes lung adenocarcinoma (LUAD) and lung squamous cell carcinoma (LUSC) as the dominant histologies. Genomic profiling of NSCLC has revealed particular patterns and frequencies of driver alterations in these subtypes, such as *TP53* (45–55%), *KRAS* (25–30%), *EGFR* (15–20%), *STK11/LKB1* (15–20%), *KEAP1* (12–17%), *NF1* (8–12%), *BRAF* (6–8%), *ALK* (3–5%) and *PTEN* (1–2%), among others, in LUAD; and *TP53* (80–90%), *CDKN2A* (45–60%), *SOX2* (20–25%), *PIK3CA* (15–25%), *PTEN* (10–15%), *NRF2* (15–20%) and *FGFR1* (10–20%), among others, in LUSC [1,2,3]. *PTEN* inactivation has functional consequences that promote a more aggressive tumor phenotype in NSCLC [4,5]. Loss of PTEN function, whether through mutation, deletion, or post-translational silencing, results in constitutive activation of the PI3K/AKT/mTOR signaling pathway, which enhances cell proliferation, survival, and motility [6]. Experimental models have shown that *PTEN*-deficient NSCLC cells exhibit increased invasiveness, epithelial-to-mesenchymal transition (EMT) features, and metastatic potential compared with *PTEN*-intact counterparts [5,6,7]. Clinically, *PTEN* alterations are associated with advanced disease stage and the presence of distant metastases at diagnosis [8]. *PTEN* loss has emerged as a determinant of immune resistance, as tumors lacking PTEN display an immunosuppressive tumor microenvironment (TME) characterized by reduced T-cell infiltration, increase in T regulatory cells, and enrichment of myeloid-derived suppressor cells (MDSCs). Our group and others have previously reported that low PTEN protein expression is associated with resistance to immune checkpoint inhibitors (ICIs) in NSCLC, leading to shorter progression-free survival (PFS) under PD-1/PD-L1 blockade [5,9]. These findings support the notion that PTEN loss not only drives tumor progression and metastasis but also confers an immunoresistant phenotype, positioning PTEN as both a prognostic and predictive biomarker in NSCLC.

Published large-scale and review series that combine mutation plus deletion and epigenetic/post-translational loss report PTEN alterations in up to ~30–40% of NSCLC patients (reviewed by [6]). Immunohistochemistry (IHC) studies consistently show much higher rates of reduced PTEN expression than sequencing-only studies. Published IHC cohorts report highly variable prevalence as follows: ~26% low PTEN in some clinical cohorts, ~46% PTEN-negative in others, and some older reports in up to ~70% patients’ tumors (reviewed by [6]). These large differences may reflect methodological variability and cohort selection.

Retrospective series and meta-analyses have tested whether decreased PTEN (by IHC or inferred from genomic data) predicts worse outcomes. Several systematic reviews and pooled analyses concluded that reduced PTEN expression is associated with worse overall survival (OS) in all NSCLC, and multiple single-center studies have linked PTEN loss with shorter progression-free survival (PFS) or relapse-free survival (RFS) as well [8,10]. However, divergent results have been published concerning the prognostic value of PTEN in LUAD and LUSC. Several studies suggest that the adverse prognostic impact of PTEN loss is more marked in LUAD, while other studies describe no histology-specific effect [11,12]. To elucidate whether the prognostic value of PTEN loss is related to LUAD or LUSC, we have evaluated PTEN protein expression by immunohistochemistry in a well-characterized cohort of NSCLC patients. In addition, databases reporting mRNA expression and mutations were interrogated with the same purpose. Finally, deconvolution analysis in PTEN-low versus PTEN-high tumors were performed to identify immune populations within the TME that may be responsible for the differential prognosis of PTEN in LUAD and LUSC. We have found that PTEN protein and mRNA loss of expression, as well as *PTEN* mutations, are consistently associated with worse outcome in LUAD, but not in LUSC. In addition, we have analyzed mutation patterns in genes of the PTEN/PI3K/AKT/mTOR pathway, as well as *PTEN* co-mutation patterns in primary tumors and metastasis. Results indicate high frequency of *PTEN-TP53* co-mutations in LUAD tumors, with increased percentage in metastasis. Evaluation of immune populations by CIBERSORT analyses identified the presence of immunosuppressive populations in PTEN-low LUAD, but not in LUSC samples. Among these populations, high tumor infiltration of M2-like macrophages in PTEN-low LUADs are indicative of poor prognosis, whereas the total number of macrophages (CD68+ cells) was related to better outcomes, reflecting the need to study further macrophage subpopulations in the context of these tumors.

## 2. Materials and Methods

### 2.1. Cohorts of Patients

The first cohort of patients to study prognosis based on immunohistochemical analysis of PTEN protein expression included 176 NSCLC patients (mainly stages I–II) diagnosed at the MD Anderson Cancer Center (MDA, Houston, TX, USA). Samples from primary tumors were collected from surgical specimens and organized in a tissue microarray (TMA). As inclusion criteria, patients with complete resection of the primary tumor without previous treatment of chemotherapy or radiotherapy were established. Reported recommendations for tumor marker prognostic studies (REMARK) criteria were followed [13]. This study was conducted according to the Declaration of Helsinki and was approved by the Institutional Review Boards and Ethical committees of MDA/UNAV (approval code 2010.111Mod5; approval date 3 February 2022). Written informed consent was obtained from each patient. Detailed clinical and pathological information of the cohort is summarized in Supplementary Table S1.

### 2.2. Immunohistochemistry

PTEN immunostaining was conducted as previously described [14]. Briefly, tissues were sectioned at 4 μm, dewaxed, and rehydrated. Antigen retrieval was performed in a RHS microwave vacuum processor (Milestone, Kalamazoo, MI, USA) with the pH6 retrieval buffer (S1699; Dako, Carpinteria, CA, USA, 5 min). Nonspecific binding sites were blocked with Vectastain Eliteprotein block (PK-6101; Vector Laboratories, Newark, CA, USA) for 20 min. Sections were incubated for 1 h with the anti-PTEN antibody (Cell Signaling Technology, Danvers, MA, USA; clone 138G6; dilution 0.13 mg/mL). Staining was visualized using Vectastain Elite secondary antibody (PK-6101; Vector Laboratories) according to the manufacturer’s instructions, followed by incubation for 10 min in diaminobenzidine (K3466; Dako). Stained TMAs were scored using a semiquantitative scoring system (0þ, negative; 1þ, weak; 2þ, moderate; and 3þ, strong). Where multiple cores were present (3–4 for some cases), all were quantified and the highest score was considered as representative of the whole tumor. Histopathological examination and quantifications were performed by two independent pathologists (CB and IW). Where discrepancies occurred, consensus was reached by joint review. For immune markers PD-1, CD68, CD3, CD4, CD8, FOXP3 and granzyme B (GzB), protocols have been previously described [15,16,17].

### 2.3. Bioinformatic and Statistical Analyses

#### 2.3.1. Data Sources and Cohort Selection

Publicly available data from the cohorts described below were accessed, and clinical data, genomic sequencing and expression data were extracted and merged using record identifiers and tumor sample barcodes. Several databases (Supplementary Table S2) were used for bioinformatic analyses: (a) TCGA-LUAD and TCGA-LUSC cohorts. Somatic mutation data for LUAD and LUSC were retrieved from The Cancer Genome Atlas (TCGA), using the R TCGAmutations package. The TCGA MC3 (Multi-Center Mutation Calling in Multiple Cancers) public MAF files were retrieved using the tcga_load() function with parameters study = “LUAD” or “LUSC” and source = “MC3”, which contains uniformly processed somatic mutation calls across TCGA datasets [18]. To reduce noise from genes that are frequently mutated due to their large size rather than functional significance, we excluded the top 100 frequently mutated large genes (FLAGSs) described in Shyr et al. [19] from all TCGA [20] analyses; (b) GENIE BPC-NSCLC cohort: data from the AACR Project GENIE Biopharma Collaborative (BPC) v2.0-public release were accessed through the Synapse platform (https://www.synapse.org; accessed on 10 January 2026) [21]; (c) lung adenocarcinoma metastasis organotropism MSK cohort [22]. Data were downloaded from cBioPortal (https://www.cbioportal.org; accessed on 22 February 2026). These two last datasets were specifically utilized to examine mutational differences between primary tumors and metastatic lesions in LUADs. For analysis of PTEN co-mutations, data from Xu et al. [20] were also retrieved. The Kaplan–Meier plotter tool for lung cancer (https://kmplot.com; accessed on 15 September 2025) and Log-rank test were used to study the prognostic value of PTEN expression in each histological subtype using the median or quartiles as cut-off values. Finally, data on PTEN expression in NSCLC patients treated with immunotherapy were accessed through dbGaP (phs002822.v1.p1).

#### 2.3.2. Quality Data Processing

Quality control filtering was applied to remove potentially artifactual variants. Mutations predicted as “benign” by PolyPhen were excluded from all analyses. Sample type filtering was performed to include only the following clinically relevant specimens: primary tumors, local recurrences, lymph node metastases, and metastases from unspecified sites. Samples from the MSK cohort were subdivided in the following two groups: (a) primary tumor-only, restricted to samples annotated as “Primary tumor”; (b) metastasis-only. Samples with unclear annotation or those derived from hematologic malignancies were excluded.

#### 2.3.3. MAF File Generation

For all cohorts, mutation data were converted to Mutation Annotation Format (MAF) using the maftools R package (version 2.26.0). Clinical annotation data were incorporated during MAF object creation to enable stratified analyses. For AACR-GENIE-BPC data [21], MAF objects were generated using the read.maf() function to accommodate the non-TCGA data structure.

#### 2.3.4. Mutation Analysis

Mutations in genes belonging to the PTEN pathway were systematically analyzed across all cohorts. The following genes were included in the pathway analysis: *PTEN*, *PIK3CA*, *AKT1*, *AKT2*, *AKT3*, *TSC1*, *TSC2* and *MTOR*. Samples harboring *PTEN* mutations were identified using the genesToBarcodes() function from maftools. Separate MAF objects were generated for *PTEN*-mutant LUAD and LUSC cohorts to characterize co-occurring mutations.

In the TCGA dataset, the top 100 frequently mutated genes were excluded from co-mutation visualization (FLAG) to focus on functionally relevant alterations; however, even after FLAG removal, no clinically meaningful co-mutation patterns were identified in *PTEN*-mutant tumors. Importantly, direct comparison of mutation frequencies across cohorts is limited by fundamental differences in sequencing methodology. While TCGA employs whole-exome sequencing, the MSK and AACR-GENIE-BPC cohorts rely on targeted oncogenic panels, which capture a predefined set of clinically relevant genes. As a result, the apparent differences in mutation prevalence across these datasets may reflect panel composition and gene length biases rather than true biological differences. With this caveat in mind, we also examined whether alterations in *PTEN* together with co-occurring mutations identified in the MSK and AACR-GENIE-BPC cohorts were detectable in the TCGA cohort as well.

#### 2.3.5. Visualization

Oncoprint visualizations were generated using the oncoplot() function from maftools. Oncoplots were generated for: PTEN pathway-specific genes with fixed gene ordering, *PTEN*-mutant subgroups displaying co-mutation patterns and primary tumor versus metastatic lesion comparisons (MSK and AACR-GENIE-BPC stratified analyses). Continuous survival curves were generated using R packages.

#### 2.3.6. Statistical Analyses

Normality of the data was assessed with the Shapiro–Wilk test. The association between PTEN expression and RFS or OS, defined as the time from the date of surgery to the date of recurrence or death, respectively, was evaluated with Kaplan–Meier curves, and significant differences among groups were assessed by the Log-rank test and logistic regression. To evaluate whether PTEN expression was an independent prognostic factor, univariable and multivariable Cox proportional regression analysis was performed using the survival package in R. Continuous survival analyses were conducted with the contsurvplot package, which implements the plot_surv_area () function to visualize how survival probability varies as a function of a continuous covariate across the follow-up period. Survival curves and summary statistics were generated with the ggsurfit and gtsummary packages. Only those variables with *p* ≤ 0.15 in the univariable analysis were included in the multivariable analysis. R and GraphPad Prism 8 (GraphPad Software Inc., San Diego, CA, USA) were used for analysis and to illustrate the results. Statistical significance was defined as *p* < 0.05 (*), *p* < 0.01 (**), and *p* < 0.001 (***).

## 3. Results

### 3.1. Low Levels of PTEN Are Associated with Poor Prognosis in Lung Adenocarcinomas, but Not in Lung Squamous Cell Carcinomas

**Figure 1:**
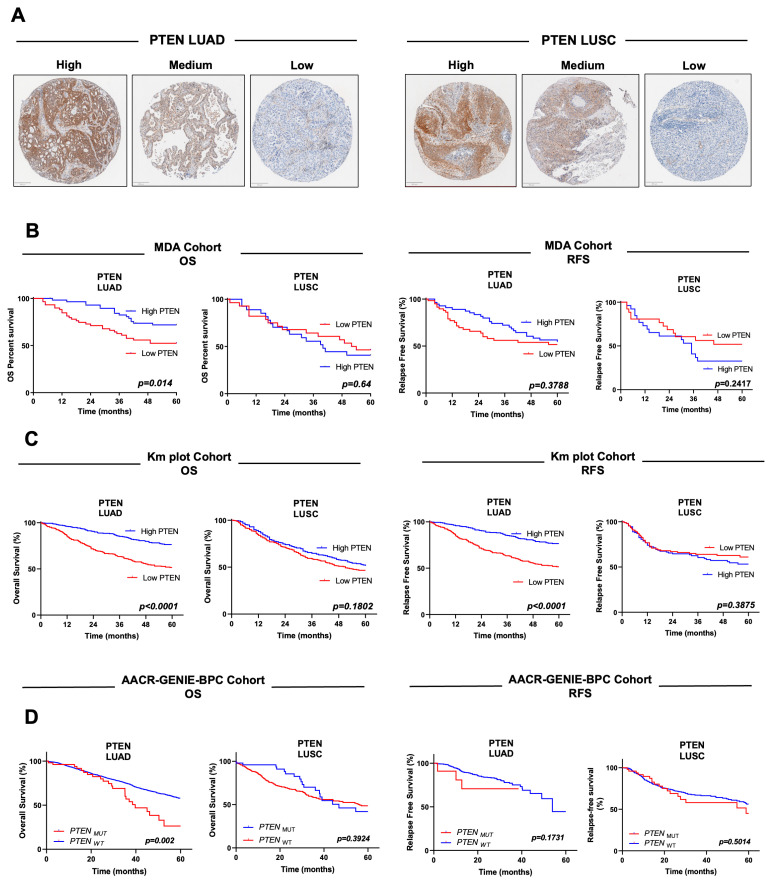
Prognostic value of PTEN protein levels, mRNA levels and mutations. (**A**) Representative images of PTEN high–medium–low immunostaining in LUAD and LUSC samples. (**B**) PTEN protein levels (MDA cohort) below the median are significantly associated with worse overall survival (OS) in LUAD, but not in LUSC. A similar trend is observed for relapse-free survival (RFS), without reaching statistical significance, in LUAD samples. (**C**) PTEN mRNA levels (Km plotter NSCLC cohort) below the median are significantly associated with reduced OS and RFS. No statistical differences based on PTEN mRNA levels are observed for LUSC. (**D**) *PTEN* mutations are significantly associated with OS in LUAD, but not in LUSC. No association is found for RFS.

To assess the prognostic value of PTEN protein levels, we first analyzed staining patterns in the TMA from the MD Anderson (MDA) cohort. The median expression value was considered as cut-off to define high versus low levels. Representative examples of tumors with high, medium and low PTEN expression in LUAD and LUSC cases are shown in Figure 1A. Kaplan–Meier curves and Log-rank tests showed that LUAD patients with low PTEN levels were significantly associated (*p* = 0.014) with worse OS. On the contrary, low PTEN levels in LUSC patients had no association with OS (Figure 1B). Low PTEN levels in LUAD tended to show worse RFS (without statistical differences), but such pattern tended to be the opposite in LUSC patients (Figure 1B).

We also evaluated the association between survival and PTEN protein expression as a continuous variable in the MDA and TCGA cohorts, using Cox proportional hazard models implemented through the survival R package. Continuous survival contours for OS and RFS were generated using the contsurvplot package (plot_survival_area function) to visualize how incremental changes in PTEN expression relate to patient outcomes. As shown in Supplementary Figure S1A,B, continuous-scale analysis in both cohorts further supports the conclusion that decreasing PTEN expression is progressively associated with poorer prognosis.

To further evaluate the prognostic significance of PTEN protein expression, we performed univariable and multivariable Cox proportional hazards analysis using median or quartiles as cut-off values in the MDA cohort. In univariable analysis performed in LUAD patients (Supplementary Table S3), low PTEN levels showed lower OS and RFS. Stratifying by the median: OS, HR: 2.17 [1.05–4.48], *p* = 0.035; RFS, HR: 1.82 [0.95–3.50], *p* = 0.06. Stratifying by quartiles (PTEN low in Q1): OS, HR: 2.77 [1.30–5.80], *p* = 0.008; RFS, HR: 2.04 [1.0–4.0], *p* = 0.040. On the contrary, no prognostic value for LUSC was found (Supplementary Table S4). For the multivariable analysis, we considered those variables whose *p*-values were significant or close to significance (*p* ≤ 0.15) in the univariable analysis. In LUAD, PTEN-low (Q1) remained as an independent indicator of worse OS (HR: 2.09 [1.02–4.30], *p* = 0.045 (Table 1)). Results on the multivariable Cox proportional hazards analysis of PTEN protein expression for LUSC patients is shown in Supplementary Table S5.

PTEN mRNA expression was next evaluated using the public KM plotter resource, which integrates The Cancer Genome Atlas (TCGA), Gene Expression Omnibus (GEO) and European Genome–Phenome Archive (EGA) datasets. Consistent with protein observations, low PTEN mRNA levels in LUAD (but not in LUSC) were significantly associated with reduced OS (*p* = 0.0001) (Figure 1C). Similar results were observed for RFS, with highly significant associations in LUAD (*p* = 0.0001), but not in LUSC (Figure 1C).

We also examined whether *PTEN* mutations correlated with patient outcomes as well (Figure 1D). In the TCGA-LUSC cohort, which includes 49 patients with *PTEN* genomic alterations, no significant association with OS (*p* = 0.3924) or RFS (*p* = 0.5014) was found. Because *PTEN* mutations are infrequent in LUAD, we aggregated survival and mutation data from seven independent cohorts (Supplementary Table S2) available through cBioportal (https://www.cbioportal.org/; accessed on 22 February 2026). This combined dataset yielded 53 LUAD patients with *PTEN* mutations. LUAD patients with *PTEN* mutations showed worse OS (*p* = 0.002). RFS showed a similar trend, without statistical difference (*p* = 0.17).

### 3.2. Co-Mutation Patterns Associated with PTEN Loss in Lung Adenocarcinomas and Lung Squamous Carcinomas

**Figure 2:**
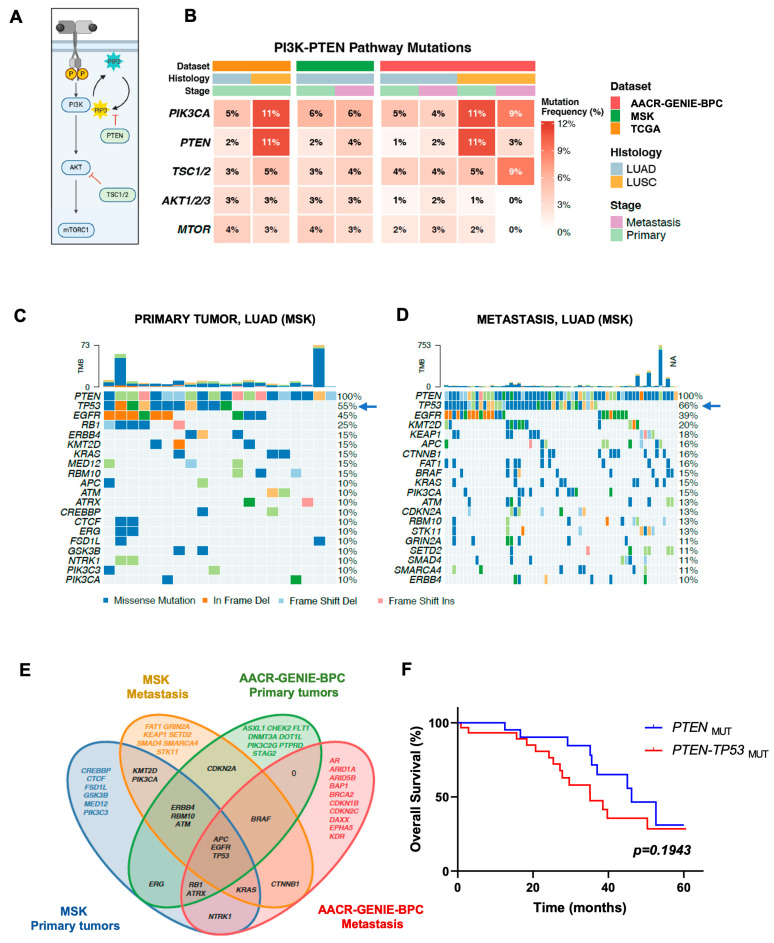
Mutation patterns associated with *PTEN* genetic alterations in LUAD/LUSC. (**A**) Representative proteins of the PTEN/PI3K/AKT/mTOR pathway, which can be mutated in either LUAD or LUSC. (**B**) Mutation frequencies in genes from the PTEN/PI3K/AKT/mTOR pathway in primary tumors and metastasis (LUAD and LUSC). In LUAD patients, the frequency of *PTEN* alterations increases in metastatic settings, which could be related to malignant progression. (**C**,**D**) Genes frequently co-mutated with *PTEN* in LUAD. *EGFR*, *TP53*, and *APC* are frequently mutated with *PTEN*, with *TP53* being highly relevant, as its co-mutation frequency is high in primary tumors (55%, arrow, (**C**)) and increases in metastasis (66%, arrow, (**D**)). MSK cohort. (**E**) Venn diagram showing overlap in mutation patterns in primary tumors and metastasis between MSK and AACR-GENIE-BPC datasets. (**F**) LUAD patients with tumors harboring *PTEN-TP53* co-mutations tend to show shorter OS compared to those having *PTEN*-mutant only tumors. NA: non-available; TMB: tumor mutational burden.

Genomic alterations in components of the PI3K/AKT/mTOR pathway occur in approximately 25–30% in NSCLC tumors [5]. Using data from TCGA, AACR-GENIE-BPC and the MSK cohorts we assessed the frequency of alterations in *PIK3CA*, *PTEN*, *TSC1*, *TSC2*, *AKT1*, *AKT2*, *AKT3* and *MTOR* across LUAD and LUSC primary tumors as well as metastases. As expected, and consistent with previous reports (reviewed in [6]), *PIK3CA* and *PTEN* mutations were particularly enriched in primary LUSC tumors (~11%) compared with primary LUAD tumors, where frequencies were lower (5–6% for *PIK3CA* and 1–2% for *PTEN*, Figure 2A,B). Alteration rates for other genes of the pathway, including *TSC1/2*, *AKT1/2/3* and *MTOR*, were comparable between both histological subtypes (Figure 2B).

Interestingly, in LUAD patients, the frequency of *PTEN* mutations increased in the metastatic setting as follows: from 2% in primary tumors to 4% in metastasis in the MSK cohort, and from 1% to 2% in the AACR-GENIE-BPC cohort. In contrast, cases of metastatic LUSC showed a decrease in the frequency of *PTEN* alterations compared with primary tumors as follows: from 11% to 9% for *PIK3CA* and from 11% to 3% for *PTEN* (Figure 2B). However, because the number of metastatic LUSCs in the AACR–GENIE-BPC cohort is small, we cannot rule out the possibility that this apparent decrease is due to insufficient statistical power.

We next sought to identify co-mutation patterns associated with *PTEN* alterations. For this analysis, we focused on MSK and AACR-GENIE-BPC cohorts, as both datasets rely on targeted mutational panels rather than whole-exome sequencing. Primary and metastatic tumors were evaluated separately to determine whether co-mutation patterns in paired samples shifted as the disease progresses. This analysis was also performed in the TCGA cohort, although the number of *PTEN*-altered LUAD cases in this dataset is small (n = 7), which may limit statistical interpretation. Mutation data from Xu et al. [20] were included in our analysis as well. Across both cohorts and both disease settings, only the following three genes were consistently co-mutated with *PTEN*: *EGFR*, *TP53*, and *APC* (Figure 2C,D and Supplementary Figure S2A–D). The overlap between co-mutations found in primary tumors and metastatic samples from MSK and AACR-GENIE-BPC cohorts is shown in Figure 2E. The frequency of *TP53* alterations increased markedly from primary tumors to metastatic samples as follows: from 55% to 66% in the MSK cohort (Figure 2C) and from 40% to 80% in the AACR-GENIE-BPC cohort (Supplementary Figure S2B). In contrast, *EGFR* mutations remained relatively stable, ranging from 45% to 39% in the MSK cohort and remaining at ~40% in the AACR-GENIE-BPC cohort, when comparing primary tumors with metastases. *APC* alterations were less frequent overall (around 10%) but showed a modest increase in the metastatic setting. These results suggest that *EGFR*, *TP53* and *APC* alterations can represent stable genomic partners of *PTEN* loss in LUAD. Moreover, the high frequency of *TP53-PTEN* co-mutations in metastasis might indicate a particularly aggressive phenotype. A Log-rank test showed that LUAD patients with tumors harboring *PTEN-TP53* mutations tended to have reduced OS compared to those harboring *PTEN*-mutant only tumors, without reaching statistical significance (Figure 2F).

Beyond this conserved core of *PTEN* co-mutated genes, each cohort and disease stage displayed unique co-alterations (Figure 2C,D and Supplementary Figure S2A–D). For instance, *KRAS-PTEN* co-mutations were found in primary tumors and metastasis of the MSK cohort but were not present in primary tumors from the AACR-GENIE-BPC dataset. In contrast, co-mutations in tumor suppressor genes such as *RBM10* and *KMT2D* appeared to be more specific to the MSK cohort. No *KMT2D* mutations were detected in the AACR-GENIE-BPC cohort, and *RBM10* mutations were only present in data from AACR-GENIE-BPC (Figure 2C,D and Supplementary Figure S2A–D). These cohort- and stage-specific differences likely reflect distinct patient populations and clinical histories, but they also underscore the substantial heterogeneity of the *PTEN*-mutant genomic context in NSCLC.

In the TCGA LUAD dataset, the small number of *PTEN*-mutant cases (n = 7) precludes robust statistical comparisons, and the co-mutation landscape showed limited overlap with those observed in the MSK and AACR-GENIE-BPC cohorts. Among these *PTEN*-altered cases, *TP53* was also the most frequently co-mutated gene (71%). However, several of the other frequent co-mutations identified in this subset, including *EPHA5* (43%), *CREBBP* (29%), *DNMT3A* (29%), and *PTPRD* (29%), were not recurrently observed in the MSK or AACR-GENIE-BPC datasets.

Finally, to explore whether PTEN expression could also play a role as a biomarker in a clinical context of immunotherapy, we accessed public RNAseq data from dbGaP (phs002822.v1.p1). Data on PTEN expression were analyzed in either immunotherapy responders or non-responders, without differentiating between LUAD and LUSC, due to the insufficient number of patients. As shown in Supplementary Figure S2E,F, a trend showing that low PTEN levels are associated with worse PFS (*p* = 0.1) and OS (*p* = 0.09) was found in NSCLC patients treated with immunotherapy who responded to the treatment. Such trend was not found in non-responder patients.

### 3.3. PTEN-Low Lung Adenocarcinomas Are Enriched in Immunosuppressive Populations

**Figure 3:**
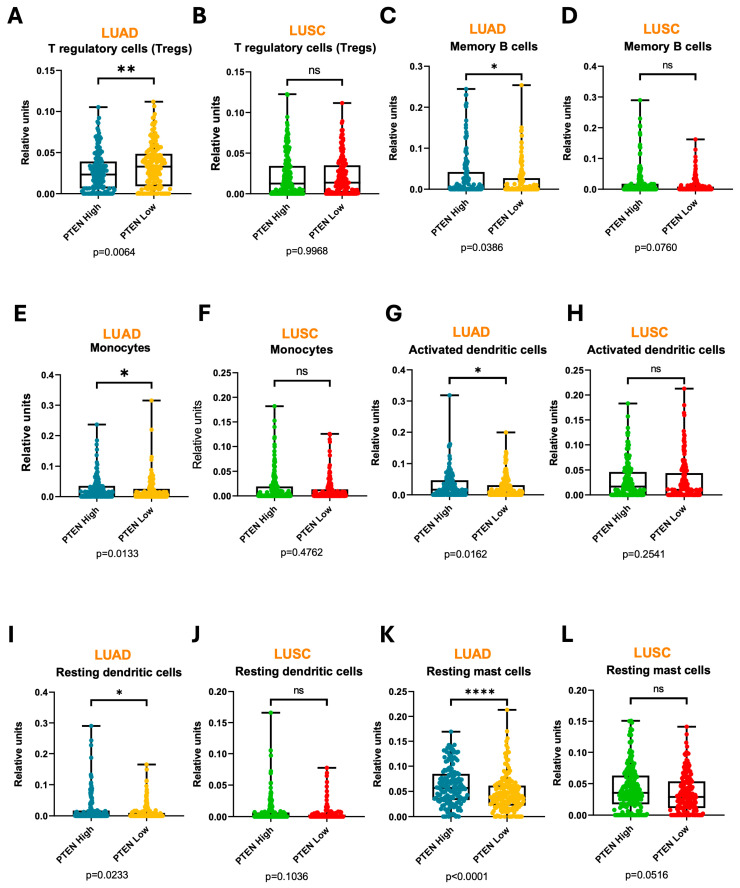
Frequency of immune populations infiltrating the tumor in LUAD or LUSC samples depending on PTEN expression, inferred by CIBERSORT. T regulatory (Treg) cells (**A**,**B**), memory B cells (**C**,**D**) and monocytes (**E**,**F**) are significantly increased in PTEN-low LUAD tumors, but not in PTEN-low LUSC tumors. Activated dendritic cells (**G**,**H**), resting dendritic cells (**I**,**J**) and resting mast cells (**K**,**L**) are significantly decreased in PTEN-low LUAD tumors, but not in PTEN-low LUSC tumors. *: *p* < 0.05; **: *p* < 0.01; ****: *p* < 0.0001; ns: not significant.

Because one of the key drivers of tumor progression and poor prognosis is an immunosuppressive TME, we estimated by bioinformatic analysis (CIBERSORT) tumor immune cell infiltration according to PTEN expression (based on median levels) (Figure 3A–L). The number of the immunosuppressive T regulatory (Treg) cells was significantly higher (*p* < 0.01) in PTEN-low LUADs than in PTEN-high LUADs, but no significant differences were found for LUSC (Figure 3A,B). In contrast, PTEN-low LUADs were significantly less enriched in proinflammatory cells, such as B memory cells (Figure 3C,D), monocytes (Figure 3E,F) and activated dendritic cells (Figure 3G,H). No significant differences were found for LUSC tumors.

The number of resting dendritic cells and resting mast cells were also lower in PTEN-low LUADs than in PTEN-high LUADs, with no differences in LUSC tumors (Figure 3I–L). The functional role of these populations in cancer is uncertain. Resting dendritic cells and resting mast cells can reflect the consequence of a prior inflammatory response, rather than ongoing inflammation. Their presence could therefore indicate resolution, exhaustion, or active suppression following earlier immune activation [23,24]. This would be in keeping with their lower abundance in PTEN-low LUAD tumors.

**Figure 4:**
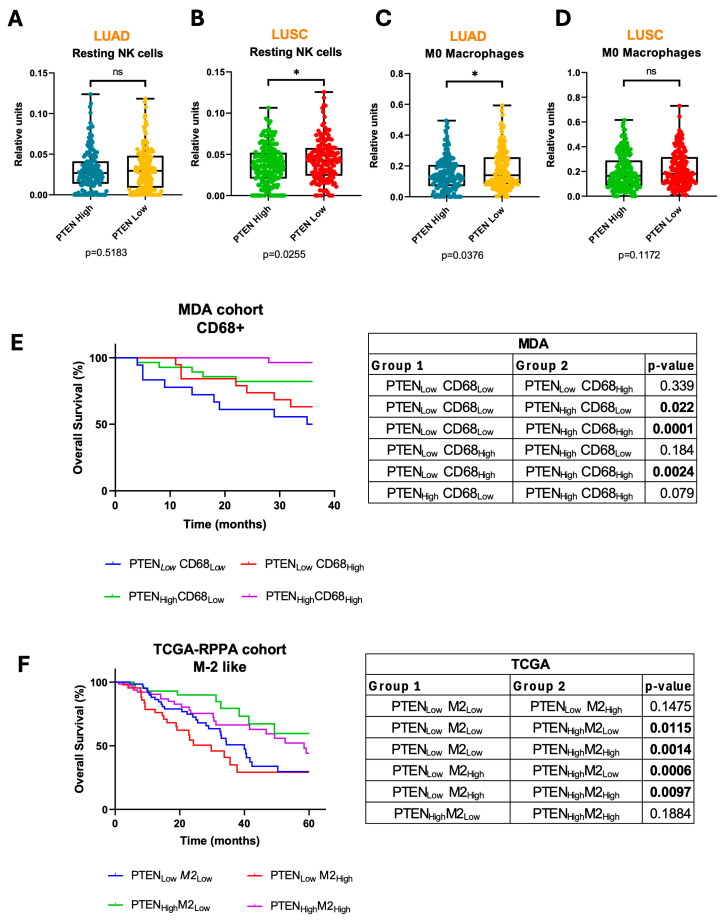
(**A**–**D**) Frequency of immune populations infiltrating the tumor in LUAD or LUSC samples depending on PTEN expression, inferred by CIBERSORT. Resting NK cells (**A**,**B**) are significantly increased in PTEN-low LUSC tumors, but not in PTEN-low LUAD tumors. M0 macrophages (**C**,**D**) are significantly increased in PTEN-low LUAD tumors but not in PTEN-low LUSC tumors. (**E**) In the MDA cohort, patients with PTEN-low tumor levels and high levels of CD68+ cells show better OS compared to those with PTEN-low levels and lower CD68+ cell infiltration. The most favorable prognosis is found for patients with PTEN-high/CD68+ high tumors. (**F**) In the TCAG-RPPA cohort, the most favorable prognosis is observed in patients whose tumors show high levels of PTEN and low levels of M2, whereas the worst prognosis is found in PTEN-low/M2-high tumors. *: *p* < 0.05; ns: not significant. *p* values in bold letters indicate statistical significance.

We also found that the number of resting NK cells (inactive) was significantly higher (*p* < 0.05) in PTEN-low LUSC than in PTEN-high LUSC specimens, with no changes in LUADs (Figure 4A,B). Tumor infiltration of M0 macrophages was significantly higher (*p* < 0.05) in PTEN-low than in PTEN-high LUADs, with no changes in LUSCs (Figure 4C,D). Previous reports have shown that both LUAD and LUSC contain substantial non-polarized M0 macrophage infiltration, but LUAD cases display an association between M0 macrophage abundance and clinical stage, which is less apparent in LUSC [25]. Higher M0 macrophage infiltration has been significantly correlated with worse prognosis in LUAD, but not in LUSC [25,26]. It has also been suggested that M0 macrophages can reflect a dysfunctional and permissive myeloid compartment [27]. Therefore, higher M0 tumor infiltration in PTEN-low compared to PTEN-high LUADs in our study might also reflect a less inflamed TME.

Other immune populations with no relevant differences between PTEN status in LUAD and LUSC are shown in Supplementary Figures S3 and S4. The number of neutrophils and resting memory CD4 T lymphocytes was increased in PTEN-high tumors, irrespective of the histological type (Supplementary Figure S3A–D). Naïve B lymphocytes were more abundant in PTEN-low than in PTEN-high LUSC tumors, whereas no differences were identified in LUAD cases (Supplementary Figure S3E,F). Some studies suggest that naïve B cell infiltration could coexist with immunosuppressive microenvironments [28], but other studies have correlated this finding with more inflamed tumors [29]. In tertiary lymphoid structures (TLSs), naïve B cells can serve as a reservoir for antigen activation, which may ultimately lead to anti-tumor immunity, but this requires maturation signals [30]. Numbers of activated NK cells, plasma cells, activated memory CD4 T lymphocytes, eosinophils and CD8 cytotoxic T lymphocytes were not different depending on PTEN status, neither in LUAD nor in LUSC (Supplementary Figures S3G–L and S4A–D).

We next conducted an univariable and multivariable analysis to study the prognostic value of immune populations in PTEN-low LUADs and PTEN-low LUSCs in the MDA cohort. In the univariable analysis, high infiltration of CD8+ (HR: 0.62 [0.39–0.96], *p* = 0.03) and C68+ (total macrophages) cells (HR: 0.47 [0.27–0.81], *p* = 0.01) in PTEN-low LUADs were significantly associated with favorable RFS. High infiltration of C68+ cells was also associated with increased OS (HR: 0.54 [0.29–0.97], *p* = 0.04) (Supplementary Table S6). Results on the univariable Cox proportional hazards analysis for PTEN-low LUSC tumors is shown in Supplementary Table S7. Multivariable analysis considering those variables from the univariable model with *p* ≤ 0.15 was then performed (Table 2). This analysis found that high levels of CD68+ cells in PTEN-low LUADs were independent factors of favorable RFS (HR: 0.44 [0.20–0.96], *p* = 0.04). In the MDA cohort, we also stratified patients by median levels of PTEN expression and CD68+ cells into the following four groups: PTEN_low_CD68_low_, PTEN_low_CD68_high_, PTEN_high_CD68_low_ and PTEN_high_CD68_high_ (Figure 4E). Patients with PTEN_high_CD68_high_ tumors showed the most favorable prognosis. Considering patients with low levels of PTEN, those with high levels of CD68+ cells had better OS compared to those with lower CD68+ cell infiltration.

In addition, the number of immunosuppressive M2-like macrophages (M2 to simplify) was inferred with CIBERSORT in the TCAG-RPPA cohort (Figure 4F). Patients were also classified by median expression into PTEN_low_M2_low_, PTEN_low_M2_high_, PTEN_high_M2_low_ and PTEN_high_M2_high_. The most favorable prognosis was found for patients whose tumors had high levels of PTEN and low levels of M2, whereas the worst prognosis was observed for PTEN_low_M2_high_ tumors. This supports that the immunosuppressive population of macrophages could be particularly relevant for the adverse outcome found in the context of dysfunctional PTEN LUAD tumors.

Considered jointly, results related to immune populations in PTEN-low LUADs suggest that the TME is highly immunosuppressive. The total number of macrophages correlates with better prognosis, whereas M0- and M2-like macrophages (immunosuppressive populations) are associated with the opposite outcome.

## 4. Discussion

*PTEN* is a central tumor suppressor gene located on chromosome 10q23 that negatively regulates the PI3K/AKT/mTOR pathway, thereby restraining cellular proliferation, survival, metabolic reprogramming, and genomic stability [4,31,32]. In NSCLC, PTEN can be altered through multiple mechanisms, including mutation, copy number loss, epigenetic silencing, and post-translational modifications [6]. Importantly, the frequency, mechanisms, and biological consequences of PTEN inactivation differ substantially between lung adenocarcinoma (LUAD) and lung squamous cell carcinoma (LUSC) [6].

Genomic data from large-scale sequencing studies indicate that *PTEN* mutations are relatively uncommon in LUAD, occurring in approximately 2–5% of cases, whereas they are more frequent in LUSC, with reported rates of ~8–15% depending on the cohort [1,2,9,33]. The Cancer Genome Atlas (TCGA) showed *PTEN* genomic alterations in ~15% of LUSC, largely driven by deletions and truncating mutations, while LUAD displayed substantially lower mutation frequencies [1,2]. However, loss of PTEN protein expression is more common than gene mutation alone would predict, highlighting the importance of non-genetic mechanisms of PTEN inactivation, including epigenetic regulation and post-translational modification [6,34]. These regulatory mechanisms are particularly relevant in smoking-associated lung cancers, where oxidative stress can directly impair PTEN phosphatase activity through reversible oxidation of its catalytic cysteine residue [35]. Therefore, functional PTEN deficiency may occur independently of genomic alterations, underscoring the importance of assessing PTEN at the protein and transcript levels when evaluating its clinical relevance.

The prognostic value of PTEN in lung cancer is promising but remains incompletely defined and appears to be histology-specific. Several prior studies have associated reduced PTEN expression with poor survival in NSCLC [10,36,37], although others have found that its prognostic significance varies depending on histological subtype [11,12]. Our results provide robust evidence indicating a subtype-specific prognostic role for PTEN. Specifically, we demonstrate across multiple independent datasets and analytical approaches that low PTEN protein and mRNA levels are consistently associated with significantly worse OS in LUAD but not in LUSC. Importantly, multivariable Cox regression analysis confirmed low PTEN levels as an independent poor prognostic factor in LUAD, reinforcing its clinical relevance beyond conventional clinicopathologic variables. Furthermore, continuous-scale modeling revealed a progressive relationship between decreasing PTEN expression and worsening patient outcomes, suggesting that PTEN functions as a quantitative determinant of tumor aggressiveness rather than a purely binary biomarker. This continuous association strengthens the biological plausibility of PTEN dosage effects in tumor progression, consistent with prior experimental studies demonstrating haploinsufficient tumor suppressor activity of PTEN, where partial loss is sufficient to promote tumorigenesis [38].

Interestingly, although *PTEN* mutations were more frequent in LUSC, confirming previous reports, no association with survival in this subtype was found. In contrast, LUAD patients harboring *PTEN* mutations showed worse survival, consistent with the prognostic impact observed at the expression level. These results may reflect differences in oncogenic dependency between LUAD and LUSC. LUAD is often driven by dominant oncogenic drivers such as *EGFR* or *KRAS*, and *PTEN* loss may synergize with these alterations to enhance oncogenic signaling and tumor progression [39]. In contrast, LUSC is characterized by widespread genomic instability and multiple concurrent pathway alterations, including frequent mutations in *TP53*, *CDKN2A*, *KEAP1* and *NFE2L2*, which may reduce the relative impact of PTEN loss alone on disease progression [2,40].

Our co-mutation analysis provides additional insight into the genomic context of *PTEN* genetic alterations. Across independent cohorts, *PTEN* mutations consistently co-occurred with *TP53*, *EGFR*, and *APC* alterations, suggesting the existence of conserved genomic partnerships. The frequent co-occurrence of *PTEN-TP53* mutations is particularly notable, as these tumor suppressors cooperate to regulate genomic stability, apoptosis, and cellular senescence. Combined disruption of *TP53* and *PTEN* has been shown to accelerate tumor progression and increase genomic instability in multiple tumor types [41]. In keeping with this concept, we observed that *TP53* alterations are more frequent in metastatic disease compared with primary tumors, with a trend towards association with worse outcome, suggesting that *TP53* loss may contribute to disease progression in the context of *PTEN* deficiency. PTEN itself also plays an important role in maintaining genomic stability through regulation of DNA repair and chromosomal integrity [42], providing a mechanistic link between PTEN loss and tumor evolution.

Co-occurrence of *EGFR* and *PTEN* alterations, observed in multiple cohorts, further underscores the biological and clinical relevance of this interaction. *PTEN* loss has been implicated as a mechanism of resistance to EGFR-targeted therapies, as it enables persistent activation of downstream PI3K/AKT/mTOR signaling despite EGFR inhibition [39]. This may contribute not only to therapeutic resistance but also to more aggressive disease behavior.

Beyond intrinsic tumor cell effects, our data indicate that PTEN dysfunctionality in LUAD is associated with substantial remodeling of the TME. PTEN-low LUAD tumors were enriched in immunosuppressive populations, including Treg cells and M0 macrophages, and showed reduced infiltration of B memory cells, monocytes, and activated dendritic cells. Treg cell enrichment is a well-established negative prognostic factor in lung cancer and contributes to immune evasion through suppression of cytotoxic T cell activity [16]. Similarly, reduced dendritic cell infiltration may impair antigen presentation and limit effective anti-tumor immune responses. The increased presence of M0 macrophages in PTEN-low LUAD tumors is also consistent with a dysfunctional or poorly activated myeloid compartment, which has been associated with worse prognosis and impaired immune surveillance [25,27]. Moreover, using inferred data from TCGA-RPPA dataset, we identified that the immunosuppressive population of M2-like macrophages was associated with worse outcome. On the contrary, our multivariable Cox regression analysis performed in the MDA cohort revealed that high levels of the CD68^+^ macrophage population (total macrophages) were independent prognostic indicators of better outcome in PTEN-low LUADs. Our data underscore the need to study macrophage subtypes in greater depth to understand their distinct roles in PTEN-low LUAD tumors.

Observations in the present study are coincident with prior reports demonstrating that PTEN loss promotes immune evasion through multiple mechanisms. PTEN-deficient tumors exhibit increased expression of immunosuppressive cytokines, reduced T-cell infiltration, and resistance to immune checkpoint blockade [5]. In NSCLC specifically, PTEN loss has been linked to reduced responsiveness to anti-PD-1 therapy and impaired anti-tumor immune activation [5]. Mechanistically, hyperactivation of PI3K/AKT/mTOR signaling in PTEN-deficient tumors can alter cytokine production, suppress interferon signaling, and promote recruitment of immunosuppressive immune populations [5]. Our findings extend these observations by demonstrating that PTEN loss is associated with a particular immunosuppressive TME (in particular M2-like macrophages) in LUAD, providing a potential explanation for the subtype-specific prognostic effects observed in our study.

In contrast, PTEN-low LUSC tumors did not exhibit the same degree of immunosuppressive remodeling, aside from an increase in resting NK cells. This difference may reflect fundamental biological differences between LUAD and LUSC. LUSC tumors typically have higher mutational burdens and greater baseline immune infiltration compared with LUAD [1,2], which may reduce the relative contribution of PTEN status to immune regulation. Alternatively, other dominant molecular alterations in LUSC may play a more prominent role in shaping the immune microenvironment.

Taken together, our results provide strong evidence that PTEN loss is a clinically and biologically significant event in LUAD, where it serves as an independent prognostic biomarker related to immunosuppressive TME. Although requiring further confirmation in larger patients’ series, *PTEN-TP53* co-mutations seem to be frequent in LUAD metastasis. Our results strongly suggest that, in the context of PTEN-low LUADs, M0- and M2-like macrophages may have an important role in conferring a malignant phenotype. In contrast, PTEN loss appears to have less prognostic and immunologic impact in LUSC, despite occurring more frequently at the genomic level. From a clinical perspective, our findings suggest that PTEN status may have potential utility for prognostic stratification and therapeutic decision-making in LUAD. Given the emerging role of PI3K/AKT/mTOR pathway inhibitors and immunotherapy combinations in lung cancer, strategies aimed at modulating immunosuppressive macrophages could be particularly relevant in the context of PTEN-low LUAD.

## Figures and Tables

**Table 1:** Multivariable Cox proportional hazards analysis of PTEN protein expression to study association with relapse-free survival (RFS) or overall survival (OS) in the MDA cohort of lung adenocarcinoma (LUAD) patients. *p* values in bold letters indicate statistical significance.

		LUAD (n = 118)					
		RFS			OS		
		HR	%95 CI	*p*	HR	%95 CI	*p*
Gender	Male				1		
	Female				1.52	(0.75–3.10)	0.247
Stage	I	1			1		
	II	0.83	(0.35–1.95)	0.668	1.92	(0.75–4.94)	0.174
	III	2.32	(1.05–5.09)	**0.036**	4.70	(1.95–11.36)	**0.001**
	IV	6.87	(1.16–40.70)	**0.034**	3.28	(0.34–31.40)	0.303
Neoadjuvant therapy	No	1			1		
	Yes	1.24	(0.46–3.35)	0.668	1.20	(0.47–3.07)	0.703
PTEN protein	Q3–Q4				1		
	Q1				2.09	(1.02–4.30)	**0.045**

**Table 2:** Multivariable Cox proportional hazards analysis of immune populations, in PTEN-low LUAD tumors, as predictors of relapse-free survival (RFS) or overall survival (OS). MDA cohort. *p* values in bold letters indicate statistical significance.

	LUAD (n = 118)					
	RFS			OS		
	HR	%95 CI	*p*	HR	%95 CI	*p*
CD4+ cells				0.76	(0.273–2.129)	0.60
CD8+ cells	0.37	(0.102–1.369)	0.14	0.88	(0.390–1.965)	0.75
CD3 + cells	2.27	(0.527–9.737)	0.27			
CD68+ cells	0.44	(0.201–0.961)	**0.04**	0.67	(0.300–1.486)	0.32
Foxp3+ cells	2.15	(0.583–7.899)	0.25			
Granzyme-b+ cells	0.65	(0.284–1.478)	0.30			

## Data Availability

The original contributions presented in this study are included in the article/Supplementary Material. Further inquiries can be directed to the corresponding author.

## Supplementary Materials

The following supporting information can be downloaded at https://www.mdpi.com/article/10.3390/medsci14020254/s1, Figure S1: Association between survival and PTEN protein expression as a continuous variable, using Cox proportional hazards models, in the MDA and TCGA cohorts; Figure S2: The seven cohorts accessed to retrieve mutation data, *PTEN* co-mutation patterns in LUAD primary tumors and metastasis, and survival analysis in NSCLC patients treated with immunotherapy; Figure S3: Frequency of immune populations infiltrating the tumor in LUAD or LUSC samples depending on PTEN expression, inferred by CIBERSORT; Figure S4: Frequency of immune populations infiltrating the tumor in LUAD or LUSC samples depending on PTEN expression, inferred by CIBERSORT; Table S1: Clinicopathological characteristics of the MDA cohort of NSCLC patients to study expression of PTEN and immune populations; Table S2: Public databases used for the study of *PTEN* mutations alone or co-mutated with other genes in LUAD and LUSC cases; Table S3: Univariable Cox proportional hazards analysis of PTEN protein expression to study association with relapse-free survival (RFS) or overall survival (OS) in the MDA cohort of lung adenocarcinoma (LUAD) NSCLC patients; Table S4: Univariable Cox proportional hazards analysis of PTEN protein expression to study association with relapse-free survival (PFS) or overall survival (OS) in the MDA cohort of lung squamous carcinoma (LUSC) patients; Table S5: Multivariable Cox proportional hazards analysis of PTEN protein expression to study association with relapse-free survival (PFS) or overall survival (OS) in the MDA cohort of lung squamous cell carcinoma (LUSC) patients; Table S6: Univariable Cox proportional hazards analysis of immune populations in PTEN-low LUAD tumors, as predictors of relapse-free survival (PFS) or overall survival (OS). MDA cohort; Table S7: Univariable Cox proportional hazards analysis of immune populations in PTEN-low LUSC tumors, as predictors of relapse-free survival (PFS) or overall survival (OS). MDA cohort.
