# Supplementary material for: Loss of PTEN as an Independent Poor Prognosis Indicator in Lung Adenocarcinoma, but Not in Squamous Cell Carcinoma, Is Associated with an Immunosuppressive Tumor Microenvironment and Distinct Co-Mutational Profiles
Source: Med Sci (Basel). 2026 May 14;14(2):254. doi: 10.3390/medsci14020254 (PMC13214857; doi:10.3390/medsci14020254)
Supplement: Supplementary file 1 [file medsci-14-00254-s001.zip › medsci-4247619-Supplementary Mateial.pdf]

Supplementary Table S1. Clinicopathological characteristics of the MDA cohort of NSCLC patients to study expression of PTEN and immune populations.

	MDA Cohort (*n*=176)	
	n (%)	
Histology	LUAD	118 (67.0)
	LUSC	58 (33.0)
Gender	Male	92 (52.3)
	Female	84 (47.7)
Age	<65	86 (48.9)
	≥ 65	90 (51.1)
Stage (IASLC)	I	79 (44.9)
	II	49 (27.8)
	III	45 (25.6)
	IIIB	1 (0.6)
	IV	2 (1.1)
Race	African or American	10 (5.7)
	Asian	4 (2.3)
	Asian or Pacific Islander	1 (0.6)
	Caucasian	156 (88.6)
	Hispanic	5 (2.8)
Tobacco history	No	16 (9.1)
	Yes	160 (90.9)
Type of smoker	Current	85 (48.3)
	Former	74 (42)
	Never	16 (9.1)
	Recent Q	1 (0.6)
Neoadjuvant therapy	No	146 (83)
	Yes	30 (17)
Adjuvant therapy	No	98 (55.7)
	Yes	77 (43.8)
	Unknown	1 (0.6)
KRAS status	Wild-type	80 (45.5)
	Mutant	35 (19.9)
	Not available	61 (34.7)

Supplementary Table S2. Public databases used for the study of *PTEN* mutations alone or co-mutated with other genes in LUAD and LUSC cases.

TCGA https://pubmed.ncbi.nlm.nih.gov/29596782/; luad_oncosg_2020
https://pubmed.ncbi.nlm.nih.gov/32015526/; luad_mskcc_2023_met_organotropism
https://pubmed.ncbi.nlm.nih.gov/37084736/; luad_mskimpact_2021
https://www.cbioportal.org/study/summary?id=luad_mskimpact_2021; luad_mskcc_2020
https://pubmed.ncbi.nlm.nih.gov/32791233/; luad_cas_2020
https://pubmed.ncbi.nlm.nih.gov/32649877/; lung_nci_2022
https://pubmed.ncbi.nlm.nih.gov/34493867/

Supplementary Table S3. Univariable Cox proportional hazards analysis of PTEN protein expression to study association with relapse-free survival (RFS) or overall survival (OS) in the MDA cohort of lung adenocarcinoma (LUAD) NSCLC patients.

		**LUAD (n=118)**					
		**RFS**			**OS**		
		**HR**	**%95 CI**	***p***	**HR**	**%95 CI**	***p***
Gender	Female	1			1		
	Male	1.17	(0.623-2.189)	0.628	1.87	(0.928-3.755)	**0.08**
Age	≥ 65	1			1		
	<65	1.29	(0.688-2.416)	0.428	1.29	(0.65-2.547)	0.469
Stage (IASLC)	I	1			1		
	II	1.14	(0.492-2.644)	0.759	2.27	(0.902-5.731)	**0.082**
	III	3.09	(1.472-6.494)	**0.003**	6.02	(2.599-13.96)	**0.00003**
	IV	12.12	(2.697-54.443)	**0.001**	4.26	(0.538-33.693)	0.170
Race	African or American	1			1		
	Asian	1.11	(0.185-6.648)	0.909	4.74E-08	(0.000-Inf)	0.997
	Asian or Pacific Islander	3.10	(0.32-30.132)	0.329	4.74E-08	(0.000-Inf)	0.998
	Caucasian	0.84	(0.257-2.746)	0.772	1.25	(0.297-5.221)	0.764
	Hispanic	2.11	(0.352-12.65)	0.414	2.00	(0.282-14.223)	0.487
Tobacco history	No	1			1		
	Yes	0.85	(0.354-2.019)	0.706	2.46	(0.589-10.29)	0.217
Type of smoker	Current	1			1		

	Former	0.93	(0.472-1.849)	0.844	1.17	(0.58-2.374)	0.656
	Never	1.13	(0.444-2.861)	0.800	0.43	(0.099-1.9)	0.268
	Recent Q	1.09E-07	(0.000-Inf)	0.997	1.07E-07	(0.000-Inf)	0.998
Neoadjuvant therapy	No	1			1		
	Yes	2.35	(1.034-5.345	**0.041**	2.27	(0.984-5.225)	**0.055**
Adjuvant therapy	No	1			1		
	Yes	0.86	(0.452-1.642)	0.650	1.00	(0.5-1.987)	0.991
KRAS status	Mutant	1			1		
	Wild-type	1.47	(0.689-3.119)	0.321	1.67	(0.722-3.863)	0.230
	Not available	2.24	(0.605-8.282)	0.227	0.86	(0.106-6.986)	0.887
PTEN protein (median)	High	1			1		
	Low	1.82	(0.95-3.50)	0.06	2.17	(1.05-4.48)	**0.035**
PTEN protein (Quartiles)	Q3-Q4	1			1		
	Q1	2.04	(1.0-4.0)	**0.040**	2.77	(1.30-5.80)	**0.008**

Supplementary Table S4. Univariable Cox proportional hazards analysis of PTEN protein expression to study association with relapse-free survival (PFS) or overall survival (OS) in the MDA cohort of lung squamous carcinoma (LUSC) patients.

		LUSC (n=58)					
		**RFS**			**OS**		
		**HR**	**%95 CI**	***p***	**HR**	**%95 CI**	***p***
Gender	Female	1			1		
	Male	1.17	(0.525-2.602)	0.703	1.49	0.6383 3.49	0.355
Age	≥ 65	1			1		
	<65	2.32	(0.926-5.827)	**0.073**	2.50	(0.931-6.701)	**0.069**
Stage (IASLC)	I	1			1		
	II	1.81	(0.558-5.889)	0.323	2.48	(0.501-12.31)	0.26515
	III	3.03	(0.961-9.551)	**0.058**	7.75	(1.763-34.06)	**0.00671**
	IIIB	18.88	(1.857-191.961)	**0.013**	14.06	(1.232-160.51)	**0.03333**
Race	African or American	1			1		
	Caucasian	1.14	(0.154-8.439)	0.898	0.96	(0.129-7.114)	0.968
	Hispanic	0.00	(0.000-Inf)	0.998	0.00	(0.000-Inf)	0.998
Tobacco history	No	1			1.00		
	Yes	25270000	(0 .000-Inf)	0.998	25060000	(0.000-Inf)	0.998
Type of smoker	Current	1			1		
	Former	1.34	(0.611-2.94)	0.465	1.73	(0.776-3.876)	0.18
	Never	0.00	(0.000-Inf)	0.998	0.00	(0.000-Inf)	0.998
Neoadjuvant therapy	No	1			1		
	Yes	1.19	(0.476-2.992)	0.706	2.09	(0.914-4.797)	**0.0805**
Adjuvant therapy	No	1			1		
	Yes	1.08	(0.491-2.362)	0.853	1.34	(0.600-2.993)	0.476
KRAS status	Mutant	1			1		
	Wild-type	0.13	(0.007-2.160)	0.153	3.70E-09	(0.000-Inf)	0.9975
	Not available	0.16	(0.0202-1.301)	**0.087**	0.16	(0.02-1.274)	**0.0835**
PTEN RNA	High	1			1		
	Low	1.14	(0.519-2.496)	0.747	1.40	(0.622-3.157)	0.415
PTEN protein (median)	High	1			1		
	Low	0.75	(0.35-1.59)	0.46	0.91	(0.41-2.03)	0.82

Supplementary Table S5. Multivariable Cox proportional hazards analysis of PTEN protein expression to study association with relapse-free survival (PFS) or overall survival (OS) in the MDA cohort of lung squamous cell carcinoma (LUSC) patients.

		**LUSC (n=58)**					
		**RFS**			**OS**		
		**HR**	**%95 CI**	***p***	**HR**	**%95 CI**	***p***
Age	<65	1			1		
	≥ 65	2.89	(1.04-8.06)	**0.042**	3.50E+00	(1.14-10.77)	**0.029**
Stage (IASLC)	I	1			1		
	II	1.99	(0.57-7.02)	0.283	2.82E+00	(0.53-14.95)	0.223
	III	3.52	(1.11-11.22)	**0.033**	7.99E+00	(1.79-35.63)	**0.006**
	IIIB	19.52	(1.86-204.51)	**0.013**	1.56E+01	(1.31-184.64)	**0.030**
Neoadjuvant therapy	No				1		
	Yes				2.44E+00	(1.01-5.89)	**0.047**
KRAS status	Mutant	1			1		
	Wild-type	0.04	(0.002-0.78)	**0.034**	6.58E-10	(0.00-Inf)	0.998
	Not available	0.05	(0.005-0.523)	**0.012**	2.71E-02	(0.002-0.31)	**0.004**
PTEN protein		1.01	(0.997-1.014)	0.170			

Supplementary Table S6. Univariable Cox proportional hazards analysis of immune populations in PTEN-low LUAD tumors, as predictors of relapse-free survival (PFS) or overall survival (OS). MDA cohort.

	**LUAD (n=118)**					
	**RFS**			**OS**		
	**HR**	**%95 CI**	***p***	**HR**	**%95 CI**	***p***
CD4+ cells	0.67	(0.357 - 1.267)	0.22	0.54	(0.266 - 1.090)	**0.09**
CD8+ cells	0.62	(0.394 - 0.965)	**0.03**	0.63	(0.385 - 1.030)	**0.07**
CD3 + cells	0.64	(0.383 - 1.055)	**0.08**	0.68	(0.384 - 1.193)	0.18
CD68+ cells	0.47	(0.270 - 0.812)	**0.01**	0.54	(0.294 - 0.975)	**0.04**
PD-1+ cells	0.69	(0.390 - 1.207)	0.19	0.73	(0.392 - 1.373)	0.33
Foxp3+ cells	0.63	(0.358 - 1.118)	**0.12**	0.63	(0.337 - 1.190)	0.16
Gzb+ cells	0.62	(0.375 - 1.035)	**0.07**	0.74	(0.423 - 1.296)	0.29
CD4/FOXP3+ cells	0.30	(0.004 - 23.270)	0.59	2.67	(0.024 - 300.100)	0.68
CD8/PD-1+ cells	2.28	(0.815 - 6.386)	**0.12**	2.09	(0.686 - 6.388)	0.19

Supplementary Table S7. Univariable Cox proportional hazards analysis of immune populations in PTEN-low LUSC tumors, as predictors of relapse-free survival (PFS) or overall survival (OS). MDA cohort.

	**LUSC (n=58)**					
	**RFS**			**OS**		
	**HR**	**%95 CI**	***p***	**HR**	**%95 CI**	***p***
CD4+ cells	0.54	(0.169 - 1.705)	0.29	1.26	(0.330 - 4.778)	0.74
CD8+ cells	0.77	(0.303 - 1.943)	0.58	1.34	(0.507 - 3.536)	0.56
CD3 + cells	0.50	(0.129 - 1.908)	0.31	0.99	(0.246 - 3.979)	0.99
CD68+ cells	1.65	(0.559 - 4.853)	0.37	2.70	(0.715 - 10.180)	0.14
PD-1+ cells	0.53	(0.168 - 1.668)	0.28	1.00	(0.281 - 3.547)	1.00
Foxp3+ cells	0.60	(0.150 - 2.364)	0.46	1.61	(0.330 - 7.825)	0.56
Gzb+ cells	0.46	(0.193 - 1.117)	**0.09**	0.76	(0.303 - 1.887)	0.55
CD4/FOXP3+ cells	0.74	(0.141 - 3.820)	0.71	0.52	(0.068 - 3.971)	0.53
CD8/PD-1+ cells	1.29	(0.168 - 9.878)	0.81	1.83	(0.203 - 16.440)	0.59

A

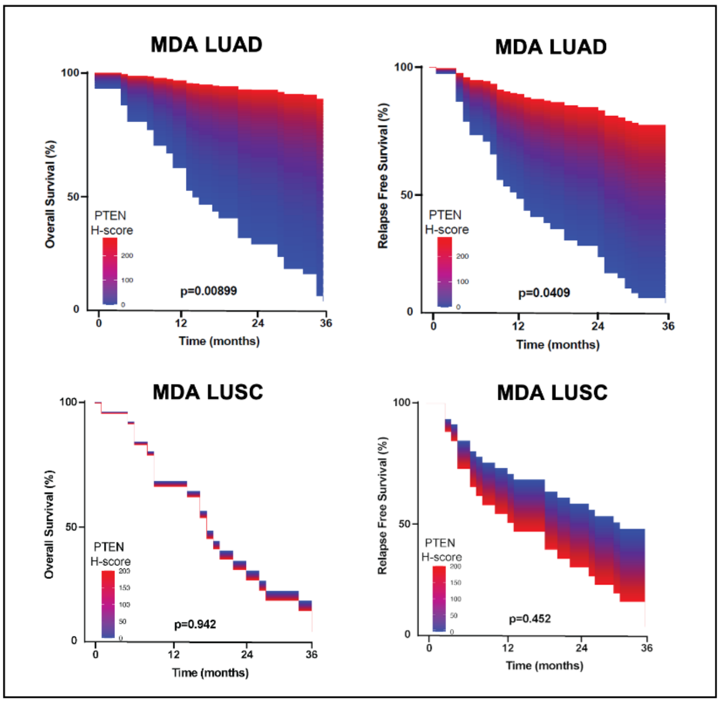

B

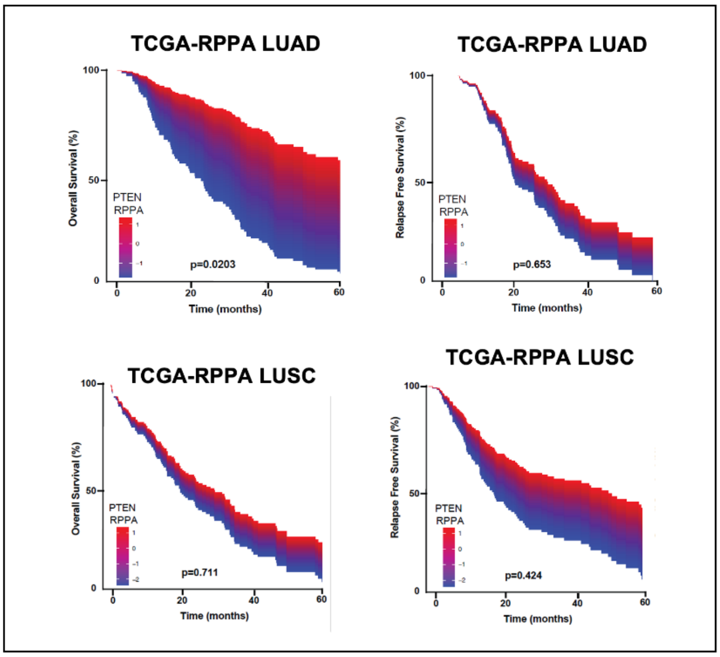

Supplementary Figure S1. Association between survival and PTEN protein expression as a continuous variable, using Cox proportional hazards models, in the MDA and TCGA cohorts. (**A**) In the MDA cohort, LUAD patients with decreasing PTEN protein levels show significantly lower RFS and OS. (**B**) In the TCGA-RPPA dataset, decreasing PTEN levels are significantly associated with OS in LUAD, with no association found for RFS. No relationship between PTEN protein levels and RFS or OS are found for LUSC.

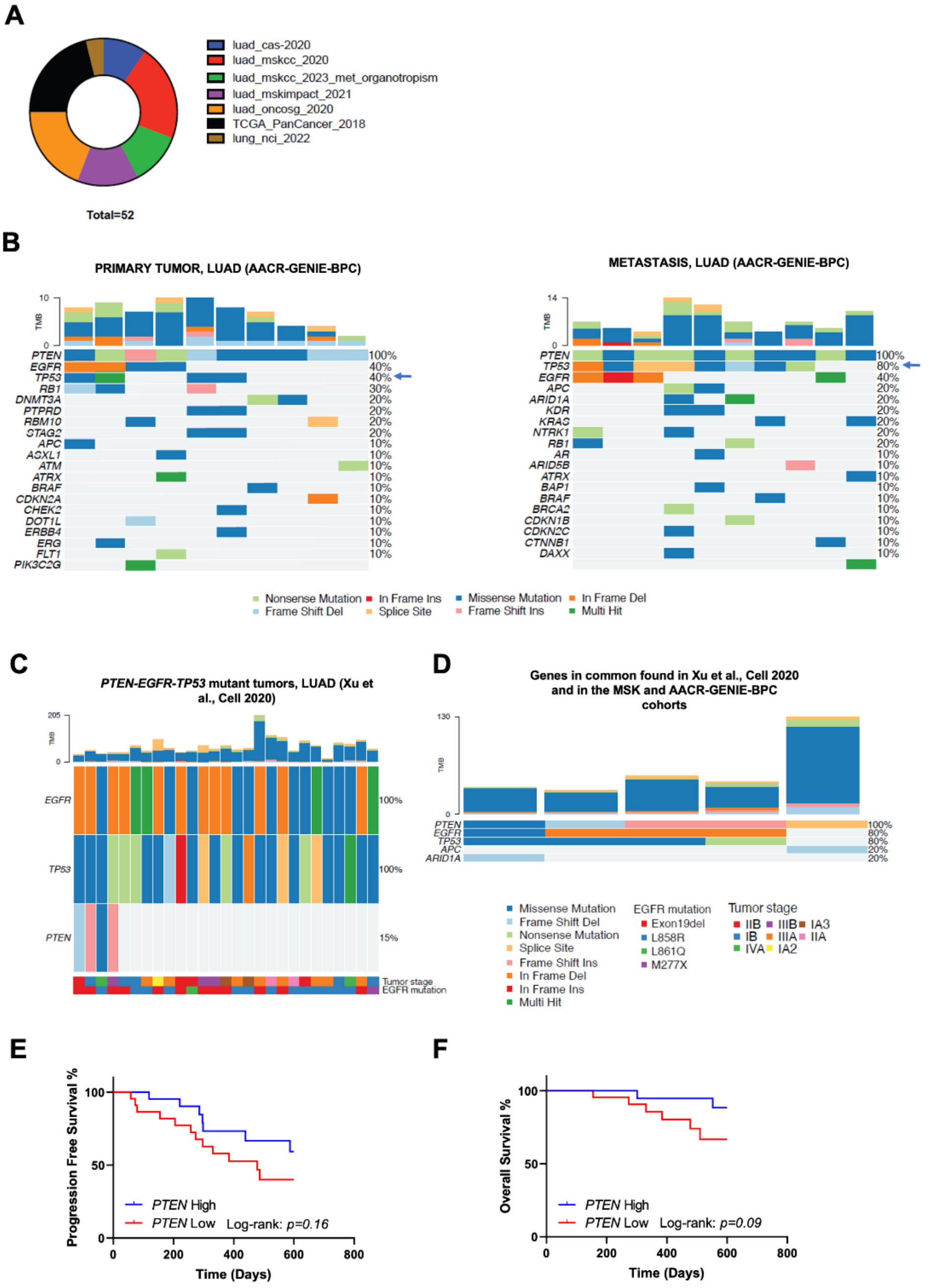

Supplementary Figure S2. (**A**) Seven cohorts were accessed to retrieve mutation data from LUAD patients. This combined dataset identified 53 LUAD patients with *PTEN* mutations. (**B**) *PTEN* co-mutation patterns analyzed in primary tumors from LUAD patients (ACCR-GENIE-BPC cohort). (**B**) *PTEN* co-mutation patterns found in metastasis from LUAD patients (ACCR-GENIE-BPC cohort). Forty percent of *PTEN* mutant LUADs also harbor *TP53* mutations (arrow). The percentage of *PTEN-TP53* co-mutations reach 80% in metastasis. (**C**) *PTEN-EGFR* and *PTEN-TP53* co-mutations in the Xu et al. dataset. (**D**) Genes commonly co-mutated with *PTEN* combining MSK and AACR-GENIE-BPC cohorts. (**E,F**) Survival analysis in NSCLC patients treated with immunotherapy. Patients were divided into responders and non-responders and PTEN mRNA levels were associated with either PFS or OS. Data were accessed from dbGaP (phs002822.v1.p1).

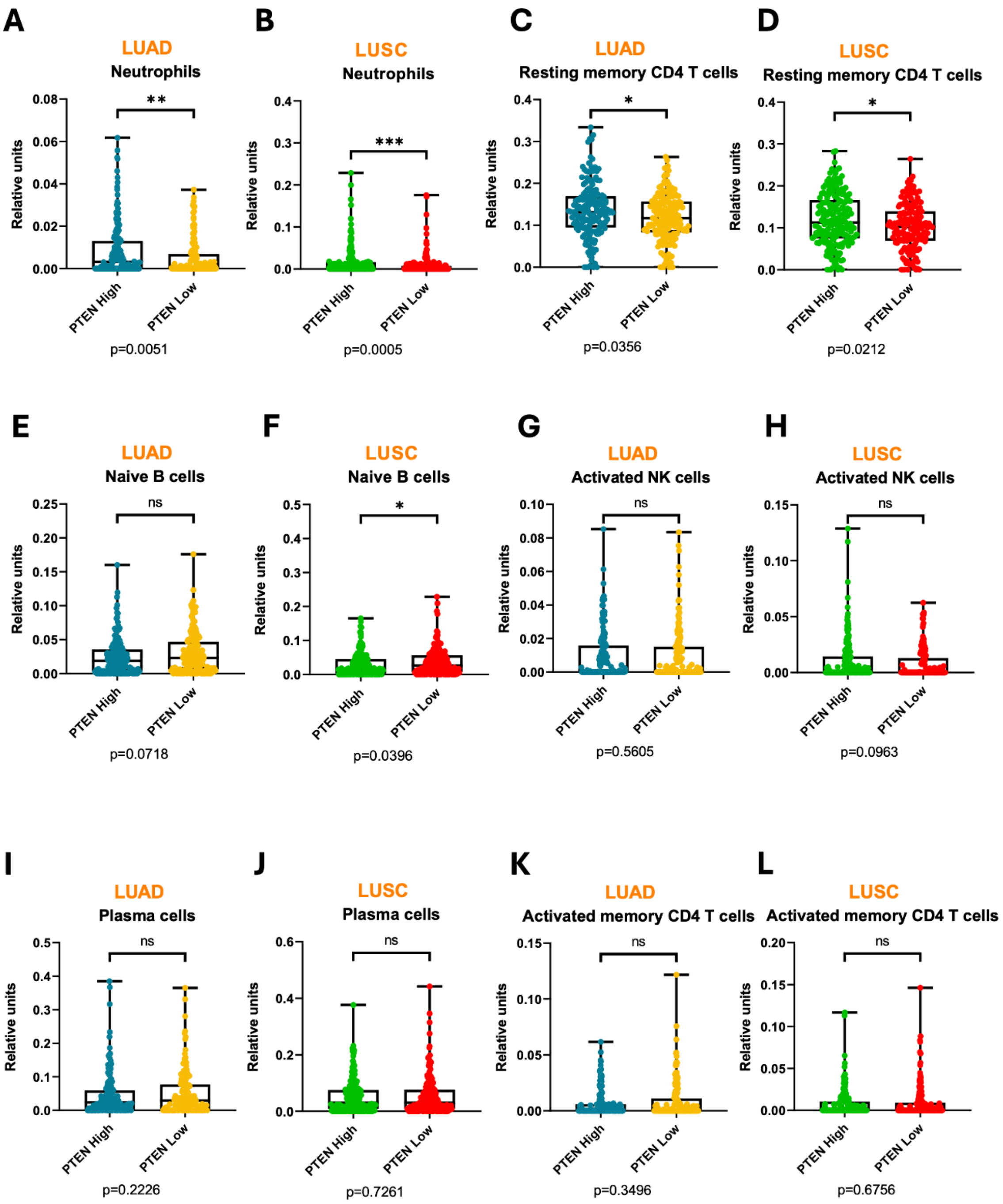

Supplementary Figure S3. Frequency of immune populations infiltrating the tumor in LUAD or LUSC samples depending on PTEN expression, inferred by CIBERSORT. Neutrophils cells (**A,B**) and resting memory CD4 T cells (**C,D**) are significantly decreased in PTEN-low LUAD and LUSC cases, compared to PTEN-high tumors. Naïve B cells are increased in PTEN-low LUSC tumors, but not in PTEN-low LUADs (**E,F**). No significant changes are observed for NK cells (**G,H**), plasma cells (**I,J**), or activated memory cells (**K,L**). *:p<0.05; **: p<0.01; ****:p<0.0001.

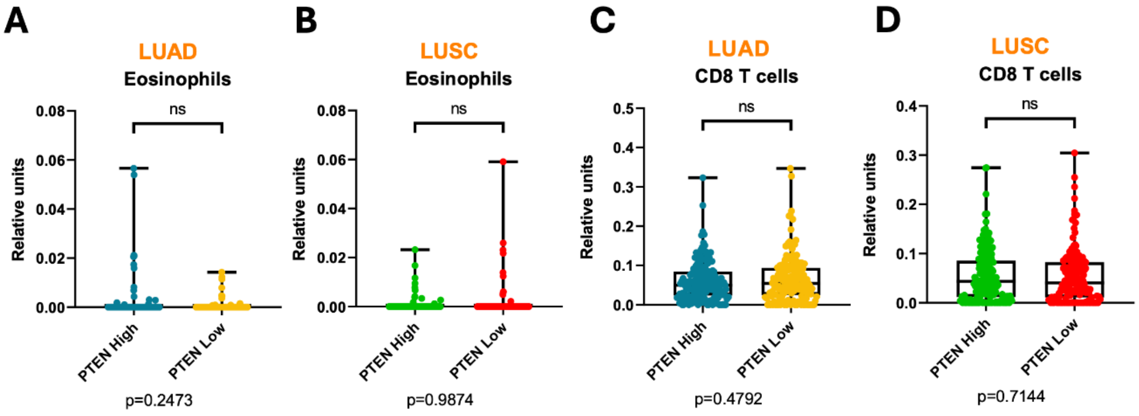

Supplementary Figure S4. Frequency of immune populations infiltrating the tumor in LUAD or LUSC samples depending on PTEN expression, inferred by CIBERSORT. Number of eosinophils (**A,B**) and CD8 T cells (**C,D**) is similar in PTEN-low LUAD and LUSC cases, compared to PTEN-high tumors.
